# Long-term mood/antidepressant effects of quetiapine extended-release formulation: an open-label, non-controlled extension study in Japanese patients with bipolar depression

**DOI:** 10.1186/s12888-019-2181-9

**Published:** 2019-06-26

**Authors:** Shigenobu Kanba, Mitsukuni Murasaki, Tsukasa Koyama, Masahiro Takeuchi, Yuriko Shimizu, Eri Arita, Kentaro Kuroishi, Masahiro Takeuchi, Shinya Kamei

**Affiliations:** 1 Department of Neuropsychiatry, Graduate School of Medical Sciences, Kyushu University, 3-1-1 Maidashi, Higashi-ku, Fukuoka, 812-8582 Japan; 2 Institute of CNS Pharmacology, 3-14-20 Sagamiohno, Minami-ku, Sagamihara, Kanagawa 252-0303 Japan; 3 Ohyachi Hospital, Clinical Research Center, 5-7-10 Ohyachi-higashi, Atsubetsu-ku, Sapporo, Hokkaido 004-0041 Japan; 4 Department of Clinical Medicine, School of Pharmacy, Kitasato University, 5-9-1 Shirokane, Minato-ku, Tokyo, 108-8641 Japan; 5 Japan/Asia Clinical Development 2, Astellas Pharma Inc.; 2-5-1 Nihonbashi-Honcho, Chuo-ku, Tokyo, 103-8411 Japan; 6 Japan-Asia Data Science, Astellas Pharma Inc, 2-5-1 Nihonbashi-Honcho, Chuo-ku, Tokyo, 103-8411 Japan; 7 Astellas Pharma Global Development, Inc, 1 Astellas Way, Northbrook, IL 60062 USA

**Keywords:** Quetiapine XR, Bipolar disorder, Depression

## Abstract

**Background:**

In an 8-week, randomized, placebo-controlled, double-blind study, an extended-release formulation of quetiapine, quetiapine XR, demonstrated efficacy and safety in Japanese patients with bipolar depression. Bipolar disorder is a chronic disease requiring continuous treatment.

**Methods:**

This was a long-term (52-week), open-label, non-controlled extension study to evaluate the long-term safety and efficacy of quetiapine XR in Japanese patients with bipolar depression who had previously completed the initial 8-week double-blind study. Efficacy was determined by the Montgomery–Åsberg Depression Rating Scale (MADRS), Hamilton Depression Scale 17-item (HAM-D_17_), and Clinical Global Impressions-Bipolar scale (CGI-BP). Safety evaluations included analysis of adverse events, clinical laboratory measures, vital signs, Drug-induced Extrapyramidal Symptoms Scale, Young Mania Rating Scale, and the Columbia Suicide Severity Rating Scale.

**Results:**

The mean (SD) MADRS total score decreased from 30.9 (6.9) at baseline to 16.1 (10.6) at week 8, and eventually to 9.1 (8.7) at week 52. The sustained efficacy of quetiapine XR treatment was also shown using HAM-D_17_ total scores, CGI-BP-Severity and Change evaluations. The most common adverse events were somnolence, nasopharyngitis, and thirst. Long-term treatment with quetiapine XR caused no substantial changes in the safety profiles, including clinical laboratory parameters, and no new safety concerns were identified.

**Conclusions:**

The efficacy of quetiapine XR was sustained long-term and no new safety concerns were identified in Japanese patients with bipolar depression.

**Trial registration:**

ClinicalTrials.gov Registration: NCT01725308. Date of registration; 12th November 2012 (retrospectively registered).

**Electronic supplementary material:**

The online version of this article (10.1186/s12888-019-2181-9) contains supplementary material, which is available to authorized users.

## Background

Bipolar disorder is a chronic mood disorder characterized by recurrent and cyclical emotional disturbances. Bipolar I disorder is a syndrome involving at least one manic episode, while bipolar II disorder involves at least one hypomanic episode and one major depressive episode [1]. According to a global survey, the lifetime prevalences of bipolar I disorder and bipolar II disorder are 0.6 and 0.4%, respectively [2].

Major treatment guidelines for bipolar disorder recommend mood stabilizers and antipsychotics as first-line therapy for the treatment of bipolar depression, and quetiapine monotherapy is recommended as one of the first-line treatments for bipolar depression [3–6].

Several clinical studies have demonstrated the efficacy of immediate-release (IR) and extended-release formulations of the atypical antipsychotic quetiapine to reduce depressive symptoms in bipolar disorder [7–9]. We recently showed in an 8-week, placebo-controlled, double-blind, parallel-group comparative study that once-daily monotherapy with 300 mg/day quetiapine XR is an effective and well-tolerated treatment for Japanese patients with bipolar depression [10].

Bipolar disorder is a long-term illness and patients with bipolar I disorder and bipolar II disorder have been shown to exhibit symptoms 47.3 and 53.9% of the time, respectively [11, 12]. Research has also shown that depressive symptoms can be approximately three times (31.9% versus 8.9%) and 39 times (50.3% versus 1.3%) longer than manic symptoms in bipolar I disorder and hypomanic symptoms in bipolar II disorder, respectively [11, 12]. Therefore, understanding the long-term efficacy and safety of the treatment in such patients is critical to further optimize the long-term management of bipolar depression with quetiapine.

The utility of long-term quetiapine treatment for patients with bipolar depression has been investigated outside Japan, and was well tolerated while showing significant reduction in the recurrence of depressive episodes [13], but it has not yet been studied in Japanese patients with bipolar depression. Therefore, the present study, an open-label, non-controlled extension study, aimed to investigate the safety and efficacy of long-term quetiapine XR treatment in Japanese patients with bipolar depression who completed the initial 8-week, double-blind study.

## Methods

### Study design

This was a multicenter, open-label, non-controlled extension study to determine the long-term safety and efficacy of quetiapine XR therapy across 98 sites. This study followed on from an 8-week placebo-controlled, double-blind, parallel-group comparative study [10] conducted in Japanese patients with bipolar depression.

**Fig. 1:**
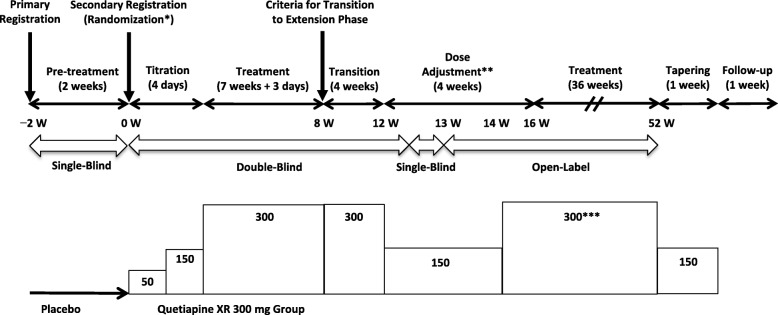
Study design. Values in boxes represent the dose of quetiapine XR in mg. *Patients were allocated to the quetiapine XR 300 mg group, the quetiapine XR 150 mg group, or the placebo group during the double-blind phase. **The dose was increased from 150 mg/day to 300 mg/day in patients who met the guideline for dose increase in week 14 or week 16. ***Dose adjustment from 300 mg/day to 150 mg/day, or vice versa, was allowed in accordance with the guideline

After completion of the initial 8-week double-blind study, patients who met the transition criteria were transferred to a 44-week long-term extension period of the study. The extension period commenced with a 4-week transition period (week 8 to week 12) followed by a 4-week dose-adjustment period (week 12 to week 16), and a continued treatment period for 36 weeks (week 16 to week 52) under open-label conditions (Fig. 1). In the initial 8-week study, patients were randomized to receive 150 mg/day quetiapine XR, 300 mg/day quetiapine XR, or placebo. The randomization to 150 mg/day quetiapine XR, however, was discontinued due to the difficulty of recruiting patients after consultation with the Pharmaceuticals and Medical Devices Agency.

### Patient population

In the initial 8-week double-blind study, patients were eligible if they were aged between 20 and 64 years; had a documented clinical diagnosis as per the Diagnostic and Statistical Manual of Mental Disorders, 4th Edition, Text Revision (DSM-IV-TR) [14] criteria for bipolar I disorder or bipolar II disorder, a recent depressive episode (296.50–296.54 or 296.89) as confirmed by the Mini-International Neuropsychiatric Interview (M.I.N.I.); HAM-D_17_ total score ≥ 20 points and HAM-D_17_ depressed mood score ≥ 2 points; and a negative pregnancy test result in female patients of childbearing potential.

Patients were not eligible if the following criteria were met: concurrent or previous history of DSM-IV-TR Axis I disorders, except bipolar disorder, within 6 months prior to informed consent; a concurrent DSM-IV-TR Axis II disorder that greatly affected the patient’s current mental status; a Young Mania Rating Scale (YMRS) total score of ≥13 points; ≥ 9 mood episodes within 12 months prior to informed consent; no response to at least two different antidepressants for > 6 weeks; history of substance or alcohol abuse; HAM-D_17_ suicide score of ≥3 points, or a history of suicide attempts within 6 months prior to informed consent.

The criteria for transition from the initial 8-week double-blind phase to the 44-week extension phase included written informed consent, negative pregnancy test, judged to be able to follow patient requirements, and the absence of any safety issues as determined by the investigators.

### Study medication

Patients who transitioned to the 4-week transition period (week 8 to week 12) continued the same dose of quetiapine XR as in the initial 8-week study (Fig. 1). After the transition period, all patients entered a 4-week dose-adjustment period (week 12 to week 16), and the dose was increased from 150 mg/day to 300 mg/day in patients who met the guideline for dose increase in week 14 or 16.

The guideline for dose increase was as follows: no moderate or severe drug-related adverse event occurred until the assessment point, and a Clinical Global Impressions-Bipolar-Change (CGI-BP-C) (Depression) rating of “no change” to “very much worse” (week 14 only). In principle, the target dose was 300 mg/day; however, if a moderate or severe drug-related adverse event occurred after the dose increase, a dose reduction to 150 mg/day was allowed. Dose adjustment from 150 mg/day to 300 mg/day, or vice versa, was allowed multiple times in accordance with the guidelines and at the discretion of the investigators. During the tapering period, quetiapine XR dosage was tapered to 150 mg/day for 1 week in patients who were administered quetiapine XR 300 mg/day at the end of treatment period, and patients were followed-up for an additional week (Fig. 1).

### Prior and concomitant medications

The following concomitant drugs were not permitted except for those specified as conditionally permitted (see next paragraph): mood stabilizers (lithium carbonate, sodium valproate), lamotrigine, antipsychotics, antidepressants, antiepileptics, antianxiety agents, hypnotics, sedatives, cytochrome P450 3A4 (CYP3A4) inhibitors or inducers, monoamine oxidase (MAO) inhibitors, psychostimulants, antiparkinsonian agents, cerebral ameliorators, antidementia agents, anorectics, and adrenaline.

Conditionally allowed concomitant drugs included lorazepam (if it had been used ≥14 days before the primary registration), only one hypnotic (zopiclone, triazolam, or eszopiclone, which had been used ≥14 days before the primary registration), and only one anticholinergic (if it had been indicated for the treatment of extrapyramidal symptoms).

### Efficacy evaluations

In the combined analysis of the double-blind treatment phase and the extension phase, the baseline was defined as the start of the double-blind treatment period (week 0).

Patients whose MADRS total score decreased by 50% or more from baseline were defined as patients with MADRS response, and patients whose MADRS total scores were ≤ 12 were defined as patients with MADRS remission. HAM-D_17_, Clinical Global Impressions-Bipolar-Severity of illness (CGI-BP-S) score and CGI-BP-C score were also assessed [15]. Patients who had a CGI-BP-C response were defined as “much improved” or “very much improved”.

Clinical assessments of MADRS, CGI-BP-S, and CGI-BP-C were conducted at weeks 10 and 12 during the transition period, weeks 13 (MADRS only), 14, and 16 during the dose-adjustment period, and weeks 18, 20, 24, 28, 32, 36, 40, 44, 48, 52, and at follow-up (week 54). Clinical assessments of HAM-D_17_ were conducted at weeks 10 and 12 during the transition period, weeks 14 and 16 during the dose-adjustment period, and weeks 20, 28, 36, 44, 52, and at follow-up (week 54).

### Safety and tolerability

Safety variables were assessed during the quetiapine XR treatment period and included adverse events (AEs), laboratory assessments (blood biochemistry, hematology, and urinalysis), body weight, vital signs (blood pressure and pulse rate), 12-lead electrocardiography (ECG) with QT interval and corrected (QTc) using Fridericia’s formula, Drug-Induced Extrapyramidal Symptoms Scale (DIEPSS), YMRS, and the Columbia Suicide Severity Rating Scale (C-SSRS).

### Statistical analyses

The target sample size was at least 100 patients treated for a period of 1 year [16].

The full analysis set included all patients who received at least one dose of quetiapine XR from commencement of the initial 8-week double-blind study. The safety analysis set included safety data from both the double-blind phase and extension phase combined, except for patients who received only placebo. For each evaluation, the measured values at each time point and summary statistics (mean, standard deviation [SD]) of changes were calculated.

Regarding the efficacy and safety assessment of the quetiapine XR 150 mg group, assignment of patients to this group was discontinued; therefore, data for this group are not presented. In addition, patients allocated to the placebo group did not receive quetiapine XR for 52 weeks, and therefore, efficacy and safety data are not shown. Therefore, the data shown here represent patients allocated to the quetiapine XR 300 mg group during the initial 8-week double-blind phase.

## Results

### Patient and disposition

**Fig. 2:**
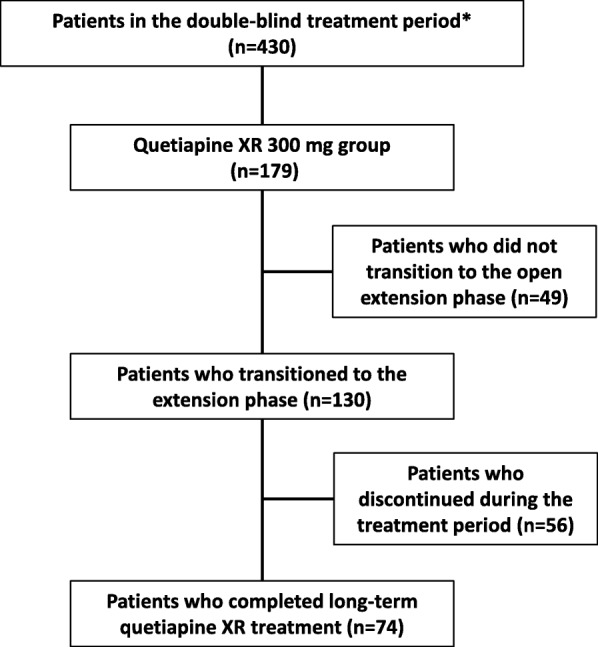
Patient disposition. *The study drug was administered to 179 patients in the quetiapine XR 300 mg group, 74 patients in the quetiapine XR 150 mg group, and 177 patients in the placebo group

The patient characteristics of the initial 8-week double-blind study were described previously [10]. Of the 179 patients receiving 300 mg quetiapine XR in the previous study, 130 patients transitioned into this extension phase. Of these 130 patients, 74 patients completed the extension phase (Fig. 2). The demographic and clinical characteristics, and the baseline values for MADRS and HAM-D_17_ evaluations are shown in Table 1.

**Table 1:** Demographic, clinical characteristics, and baseline values for MADRS and HAM-D_17_ evaluations

Variable		Quetiapine XR (*n* = 179)
Age (Years), mean (SD)		38.1 (11.2)
Sex	Male	86 (48.0%)
	Female	93 (52.0%)
Diagnosis	Bipolar I Disorder	51 (28.5%)
	Bipolar II Disorder	128 (71.5%)
Number of Mood Episodes in the Past 12 Months	≥4	14 (7.8%)
MADRS Total Score, mean (SD)		30.9 (6.9)
HAM-D_17_ Total Score, mean (SD)		23.0 (3.0)

n (%)

*MADRS* Montgomery–Åsberg Depression Rating Scale, *HAM-D*_*17*_ Hamilton Depression Scale Item-17

### Efficacy

#### MADRS

**Fig. 3:**
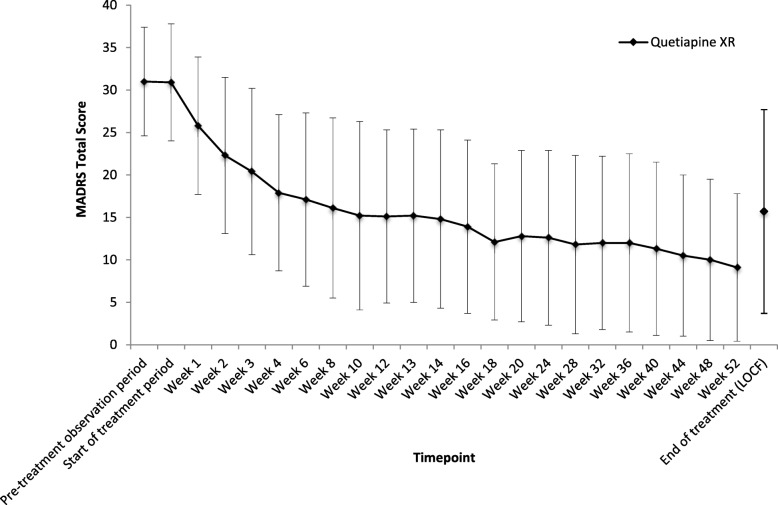
Time course of mean MADRS total score**.** Observed cases are shown. *MADRS* Montgomery–Åsberg Depression Rating Scale, *LOCF* Last Observation Carried Forward

The mean (SD) MADRS total score in the observed cases (OC) decreased from 30.9 (6.9) at baseline to 16.1 (10.6) at week 8, and the decrease continued thereafter to 9.1 (8.7) at week 52 (Fig. 3). The mean (SD) change from baseline to the end of treatment was − 15.2 (12.2).

The proportion of patients with a MADRS response or MADRS remission at the end of treatment is shown in Table 2. The proportion of patients with a MADRS response (OC) increased over time until week 12 (56.7%), and subsequently tended to increase gradually until week 52 (77.0%). At the end of the treatment, it was 49.7%. The proportion of patients with a MADRS remission (OC) increased over time until week 18 (59.1%), and subsequently remained within a range of 55.7% (week 20) to 68.9% (week 52). At the end of the treatment, it was 45.8%.

**Table 2:** MADRS response, MADRS remission, and CGI-BP-C response at the end of treatment

		Quetiapine XR (n = 179)
Patients with a Response Based on MADRS Total Score		89 (49.7%)
Patients with a Remission Based on MADRS Total Score		82 (45.8%)
Patients with a Response Based on CGI-BP-C Score	Overall Bipolar Illness	92 (51.4%)
	Depression	95 (53.1%)
	Mania	0 (0.0%)

n (%)

*MADRS* Montgomery–Åsberg Depression Rating Scale, *CGI-BP-C* Clinical Global Impressions-Bipolar-Change

Subgroup analyses of patients stratified by sex, age, diagnosis (bipolar I or bipolar II disorder), baseline MADRS total score, and baseline HAM-D_17_ total score were performed for the change from baseline in MADRS total score, revealing no clear differences between subgroups based on sex, age, and diagnosis. In the subgroups stratified by the baseline MADRS total score and the baseline HAM-D_17_ total score, the change from baseline in MADRS total score tended to be greater in the subgroup with the more severe symptoms.

#### HAM-D_17_

The mean (SD) HAM-D_17_ total score (OC) decreased from 23.0 (3.0) at baseline to 11.5 (6.6) at week 8, and this decrease continued thereafter to 7.1 (6.1) at week 52. The mean (SD) change from baseline at the end of the treatment was − 11.6 (8.3).

#### CGI-BP-S

The mean (SD) CGI-BP-S (Overall bipolar illness) score (OC) decreased from 4.4 (0.8) at baseline to 3.0 (1.1) at week 8, and that at week 52 was 2.1 (1.1). The mean (SD) change from baseline at the end of treatment was − 1.5 (1.5). The mean (SD) CGI-BP-S (Depression) decreased (OC) from 4.5 (0.7) at baseline to 3.0 (1.1) at week 8, and that at week 52 was 2.1 (1.1). The mean (SD) change in CGI-BP-S (Depression) score from baseline at the end of treatment was − 1.6 (1.5).

#### CGI-BP-C

The proportion of CGI-BP-C (Overall bipolar illness and Depression) responders at the end of treatment is described in Table 2. The proportion of patients with a CGI-BP-C (Overall bipolar illness) response (OC) increased over time until week 14 (62.7%), and subsequently remained within a range of 59.8% (week 16) to 78.4% (week 52). At the end of treatment, it was 51.4%. The proportion of patients with a CGI-BP-C (Depression) response (OC) also increased over time until week 14 (62.7%), and remained within a range of 59.8% (week 16) to 78.4% (week 52). At the end of treatment, it was 53.1%.

### Safety and tolerability

#### Adverse events

Table 3 provides a summary of AEs. The incidence of AEs was 95.5% (171/179 patients), and the incidence of drug-related AEs was 88.8% (159/179 patients). No deaths were reported, and serious adverse events (SAEs) were reported in five patients (spinal compression fracture, mania, appendicitis, and atrial flutter in one patient each, asthma and altered state of consciousness in one patient). The incidence of AEs leading to discontinuation was 28.5% (51/179 patients). Most of the AEs were mild or moderate in severity; three severe AEs occurred in two patients (mania in one patient and asthma and altered state of consciousness in one patient).

**Table 3:** Summary of adverse events

	Quetiapine XR (*n* = 179)	
	Number of Patients (%)	Number of AEs
AEs	171 (95.5%)	845
Drug-related AEs	159 (88.8%)	559
Deaths	0	–
SAEs	5 (2.8%)	6
Drug-related SAEs	2 (1.1%)	2
AEs leading to discontinuation	51 (28.5%)	64
Drug-related AEs leading to discontinuation	39 (21.8%)	49

*AEs* adverse events, *SAEs* serious adverse events

Additional file 1 Table S1 summarizes AEs that occurred in > 5% of patients during this study. The most common AE was somnolence (54.2%), followed by nasopharyngitis (32.4%), and thirst (28.5%). AEs with an incidence of 10% or higher were malaise (12.3%), constipation (11.7%), blood prolactin increased (11.7%), akathisia (11.7%), and weight increased (10.6%).

Regarding the period during which first onset of AEs was observed, the incidence of AEs was the highest (55.9%) during the early stage of treatment (Day 1–7), with the incidence in subsequent periods ranging from 0 to 10.7%.

#### AEs related to manic and hypomanic symptoms

The incidence of AEs related to manic or hypomanic symptoms was 3.9% (7/179 patients); these were hypomania (2.8%, 5/179 patients), bipolar I disorder (0.6%, 1/179 patients), and mania (0.6%, 1/179 patients). Manic symptoms, as assessed by the mean (SD) YMRS total score, were 2.1 (1.7) at baseline and 1.7 (4.3) at the end of treatment, showing no worsening in the mean YMRS total score.

#### AEs related to extrapyramidal symptoms

The incidence of AEs related to extrapyramidal symptoms was 19.0% (34/179 patients). The most common AE was akathisia (11.7%, 21/179 patients); however, there was little change in DIEPSS during the treatment period.

#### AEs related to suicide

The incidence of AEs related to suicide was 4.5% (8/179 patients); these events included intentional self-injury (1.1%, 2/179 patients), suicidal ideation (1.1%, 2/179 patients), suicide attempt (1.1%, 2/179 patients), and self-injurious behavior (1.1%, 2/179 patients).

#### Withdrawal symptoms and rebound phenomenon

To assess drug withdrawal syndrome, withdrawal symptoms were assessed among the AEs that occurred during the dose-tapering period and the follow-up period. The incidence of AEs was 13.6% (9/66 patients) in the dose-tapering period and 18.8% (18/96 patients) in the follow-up period. Of these AEs, drug withdrawal syndrome was reported in two patients (2.1%) in the follow-up period, and withdrawal syndrome was reported in one patient (1.5%) in the dose-tapering period and in two patients (2.1%) in the follow-up period. These AEs resolved, except for withdrawal syndrome in one patient.

Regarding rebound phenomenon, there was no obvious worsening observed in the follow-up period in each assessment of depression symptoms, including MADRS.

#### Clinical laboratory evaluations

Changes in clinical laboratory evaluations are presented in Table 4. The incidence of weight increased was 10.6%, and the mean (SD) change from baseline in body weight at the end of treatment was 1.02 (3.99) kg. No particularly significant changes in the mean values were found for any of the hematological parameters. For blood biochemistry parameters, the mean triglyceride levels tended to increase from baseline to the end of treatment (128.2 mg/dL at baseline and 139.9 mg/dL at the end of treatment). There were no significant changes in the mean values for blood glucose, HbA1c, total cholesterol, and blood prolactin measures from baseline to the end of treatment.

**Table 4:** Clinical laboratory evaluations

	Baseline	End of treatment	Change from baseline
	Quetiapine XR	Quetiapine XR	Quetiapine XR
	Mean (SD)	Mean (SD)	Mean (SD)
Body weight (kg)	63.15 (13.71)	64.26 (13.67)	1.02 (3.99)
Blood glucose (mg/dL)	98.5 (14.2)	100.3 (16.4)	2.1 (18.4)
HbA1c (%)	5.05 (0.27)	5.09 (0.31)	0.04 (0.23)
Total Cholesterol (mg/dL)	191.0 (35.6)	193.7 (38.3)	3.2 (29.3)
HDL-C (mg/dL)	56.5 (16.7)	56.5 (16.8)	0.1 (8.2)
LDL-C (mg/dL)	115.4 (32.0)	115.8 (33.2)	0.6 (24.1)
Triglycerides (mg/dL)	128.2 (80.1)	139.9 (116.6)	11.8 (90.2)
Prolactin (ng/mL)	10.529 (6.779)	10.040 (6.472)	−0.458 (7.002)

*HbA1c* Hemoglobin A1c, *HDL-C* High-density lipoprotein-cholesterol, *LDL-C* Low-density lipoprotein-cholesterol

The 12-lead ECG findings showed that four patients had clinically significant abnormalities as judged by an investigator. Of these patients, one patient had a clinically significant abnormality (atrial flutter) from week 8 through to week 28 that was assessed as a SAE and subsequently underwent catheter ablation. The mean change (SD) from baseline in QTc (Fridericia) was 1.9 (13.2) msec at the end of treatment, and none of the patients had QTc (Fridericia) exceeding 480 msec at any assessment point.

## Discussion

Previous long-term studies, EMBOLDEN I and II, have showed that quetiapine monotherapy in patients with bipolar depression was effective within 8 weeks, and the efficacy for 52 weeks as assessed by the change in MADRS total score [13, 17, 18]. In these studies, the risk of recurrence of a depressive relapse was significantly lower with quetiapine compared with placebo, which is suggestive of quetiapine’s efficacy in both short- and long-term therapy in patients with bipolar depression. Moreover, these studies showed that quetiapine monotherapy has an acceptable safety and tolerability profile.

This open-label extension study evaluated the long-term safety and efficacy of quetiapine treatment in Japanese patients with bipolar depression who had completed the initial 8-week double-blind study [10]. During the acute treatment period, administration of 300 mg/day quetiapine XR for 8 weeks resulted in a superior reduction from baseline in MADRS total score in comparison with placebo. For those patients that successfully transitioned to long-term treatment, the efficacy of quetiapine XR treatment was maintained as evidenced by rating MADRS, HAM-D_17_, CGI-BP-S, and CGI-BP-C in a patient population that included both bipolar I and bipolar II disorder diagnoses. Furthermore, there were no marked differences in the development of AEs after long-term quetiapine XR administration, and no new safety concerns in terms of laboratory values or vital signs. The number of patients whose dose of quetiapine XR was increased from 150 mg/day to 300 mg/day during the dose-adjustment period was 98. Of these 98 patients, 68 maintained a dose of 300 mg/day for the rest of the extension phase, and quetiapine XR monotherapy for bipolar depression was well tolerated.

Changes of body weight, metabolic parameters, and blood prolactin levels are common after the administration of atypical antipsychotics [19–21]. In this study, long-term quetiapine XR treatment caused an increase in mean weight; however, this increase in weight did not cause patients to withdraw from the study. Blood glucose and HbA1c levels showed an increase; however, this increase was not significant. Serum triglyceride levels also showed a tendency to increase. Mean increases in total cholesterol and triglycerides levels have previously been reported for both quetiapine and olanzapine, with regular monitoring of metabolic parameters recommended as routine clinical practice [22].

A number of typical and atypical antipsychotics have demonstrated induction of a sustained hyperprolactinemia above normal ranges [23]. However, quetiapine has previously been documented to decrease blood prolactin levels [24, 25]. In this long-term study, there was no significant change in the mean change from baseline in blood prolactin levels.

The safety profile of quetiapine in patients with bipolar depression is well established in clinical trials [7–9, 13, 17, 18]. In this extension study, no deaths were reported, and all AEs were mild to moderate in severity, except for three severe AEs. In this instance, quetiapine XR treatment in patients with severe AEs was discontinued, and most AEs were successfully resolved. Therefore, in summary, the safety results described here are in line with previous observations.

Patients with bipolar depression are vulnerable to drug-induced extrapyramidal symptoms with typical antipsychotic agents [26]. However, quetiapine is an atypical antipsychotic that comes with a lower risk for acute extrapyramidal symptoms [27], and this was confirmed in this study. The long-term administration of quetiapine XR monotherapy in patients with bipolar depression showed no particularly significant trends of drug-induced extrapyramidal symptoms as confirmed using DIEPSS.

Patients with bipolar depression are also vulnerable to treatment-induced manic switching, particularly in those treated with antidepressants monotherapy [28]. Quetiapine monotherapy reduces bipolar depressive symptoms in the absence of worsening mania symptoms, as evidenced in this study by the lack of worsening in the mean YMRS total score.

Drug-induced withdrawal syndrome and withdrawal syndrome was observed in some patients, but the incidence was not high, and, for the majority of those patients, AEs were resolved. Assessment of rebound phenomenon showed no obvious worsening in any of the efficacy variables during the post-treatment observation period, including MADRS analysis. Although depression and AEs related to suicide were reported during the dose-tapering period and the follow-up period, all of these AEs were non-serious and confirmed to have been resolved.

The present study has some limitations. The results of this long-term study include data on quetiapine XR treatment for 52 weeks. However, the initial 8-week-period was a double-blind study, and the remaining extension phase was an open-label study; therefore, the manner of blinding was different in different parts of this study. Furthermore, the study design was changed because allocation of patients to the quetiapine XR 150 mg group was discontinued during the double-blind phase. In addition, patients who were allocated to the placebo group in the double-blind study did not receive quetiapine XR for 52 weeks. Therefore, data from patients allocated to the quetiapine XR 300 mg group in the double-blind study were used to show the long-term efficacy and safety of quetiapine XR for bipolar depression. Additionally, these findings in Japanese patients may not be generalizable to other populations.

## Conclusions

This study was conducted to evaluate the efficacy and safety of quetiapine XR in Japanese patients with bipolar depression over a 44-week extension treatment period to an 8-week double-blind study. The long-term efficacy and safety of treatment with quetiapine XR in Japanese patients with bipolar depression were confirmed. The efficacy of quetiapine XR was sustained until week 52, which was the final assessment in the treatment period, for all the efficacy variables. AEs, including somnolence, thirst, and various other abnormal laboratory values, were observed; however, these safety profiles have already been confirmed in previous quetiapine XR trials. Therefore, no new safety concerns were found after long-term administration.

## Additional file

Additional file 1: **Table S1**: Adverse events that occurred in >5% of patients. (PDF 99 kb)

## Data Availability

The datasets used and/or analyzed during the current study are available on reasonable request from https://www.clinicalstudydatarequest.com/. The final report is available on the Astellas Clinical Study Results (ACSR) website: https://astellasclinicalstudyresults.com/hcp/compoundresult.aspx?PC=31

## Abbreviations

CGI-BP-C: Clinical Global Impressions-Bipolar-Change
CGI-BP-S: Clinical Global Impressions-Bipolar-Severity of illness
C-SSRS: Columbia Suicide Severity Rating Scale
DIEPSS: Drug-induced extrapyramidal symptoms scale
DSM-IV-TR: Diagnostic and Statistical Manual of Mental Disorders, 4th Edition, Text Revision
ECG: Electrocardiography
HAM-D_17_: Hamilton Depression Scale 17-item
IR: Immediate Release
M.I.N.I: Mini-International Neuropsychiatric Interview
MADRS: Montgomery–Åsberg Depression Rating Scale
MAO: Monoamine oxidase
YMRS: Young Mania Rating Scale
