# Supplementary material for: Long-term mood/antidepressant effects of quetiapine extended-release formulation: an open-label, non-controlled extension study in Japanese patients with bipolar depression
Source: BMC Psychiatry. 2019 Jun 26;19:198. doi: 10.1186/s12888-019-2181-9 (PMC6595609; doi:10.1186/s12888-019-2181-9)
Supplement: Supplementary file 1 — Table S1: Adverse events that occurred in >5% of patients. (PDF 99 kb) [file 12888_2019_2181_MOESM1_ESM.pdf]

Supplemental Table

Table S1: Adverse events that occurred in >5% of patients

	**Quetiapine XR (n=179)**	
	Number of Patients	Percentage of Patients
**GI Disorders**	**63**	**35.2**
Constipation	21	11.7
Nausea	10	5.6
**General Disorders and Administration Site Conditions**	**71**	**39.7**
Malaise	22	12.3
Thirst	51	28.5
**Infections and Infestations**	**79**	**44.1**
Gastroenteritis	9	5.0
Nasopharyngitis	58	32.4
**Investigations**	**75**	**41.9**
ALT increased	13	7.3
Blood creatine phosphokinase increased	13	7.3
Blood prolactin increased	21	11.7
Blood triglycerides increased	9	5.0
Weight increased	19	10.6
**Nervous Systems Disorders**	**115**	**64.2**
Akathisia	21	11.7
Dizziness postural	9	5.0
Headache	12	6.7
Somnolence	97	54.2

GI gastrointestinal, *ALT* alanine aminotransferase, *AST* aspartate aminotransferase
